# Synthesis of New Styrylquinoline Cellular Dyes, Fluorescent Properties, Cellular Localization and Cytotoxic Behavior

**DOI:** 10.1371/journal.pone.0131210

**Published:** 2015-06-26

**Authors:** Marzena Rams-Baron, Mateusz Dulski, Anna Mrozek-Wilczkiewicz, Mateusz Korzec, Wioleta Cieslik, Ewelina Spaczyńska, Piotr Bartczak, Alicja Ratuszna, Jaroslaw Polanski, Robert Musiol

**Affiliations:** 1 A. Chelkowski Institute of Physics, University of Silesia, Uniwersytecka 4, Katowice, 40–007, Poland; 2 Silesian Center for Education and Interdisciplinary Research, 75 Pulku Piechoty 1A, Chorzow, 41–500, Poland; 3 Institute of Chemistry, University of Silesia, Szkolna 9, Katowice, 40–006, Poland; 4 Institute of Material Sciences, University of Silesia, 75 Pulku Piechoty 1A, Chorzow, 41–500, Poland; University of East Anglia, UNITED KINGDOM

## Abstract

New styrylquinoline derivatives with their photophysical constants are described. The synthesis was achieved *via* Sonogashira coupling using the newly developed heterogeneous nano-Pd/Cu catalyst system, which provides an efficient synthesis of high purity products. The compounds were tested in preliminary fluorescent microscopy studies to in order to identify their preferable cellular localization, which appeared to be in the lipid cellular organelles. The spectroscopic properties of the compounds were measured and theoretical TD-DFT calculations were performed. A biological analysis of the quinolines that were tested consisted of cytotoxicity assays against normal human fibroblasts and colon adenocarcinoma cells. All of the compounds that were studied appeared to be safe and indifferent to cells in a high concentration range. The presented results suggest that the quinoline compounds that were investigated in this study may be valuable structures for development as fluorescent dyes that could have biological applications.

## Introduction

Quinoline-based compounds are of special interest to organic and medical chemists due to their variety of applications, which are not limited only to pharmaceutical uses. Such compounds, which have extended delocalized π-electron systems, may exhibit exciting optical properties that may offer opportunities for potential applications such as optical materials or luminescent probes. Styrylquinolines are an excellent example of such applicable and biologically relevant materials. They are known antiviral agents that are a styrylquinoline inhibitors of HIV integrase [1], antifungal [2] and anticancer agents [3]. Particular attention has been paid to this class of compounds because of their electroluminescence and photochromic properties, which could permit their potential utility in optical devices or as biosensors [4,5]. Many reports have pointed out the feasible use of molecules such as fluorophores that could be suitable for staining intracellular components such as nucleic acids or proteins [6,7]. Recently, fluorescent styrylquinoline was reported as an application as an amyloid imaging sensor, which could be useful in diagnosing Alzheimer’s disease [8].

**Fig 1:**
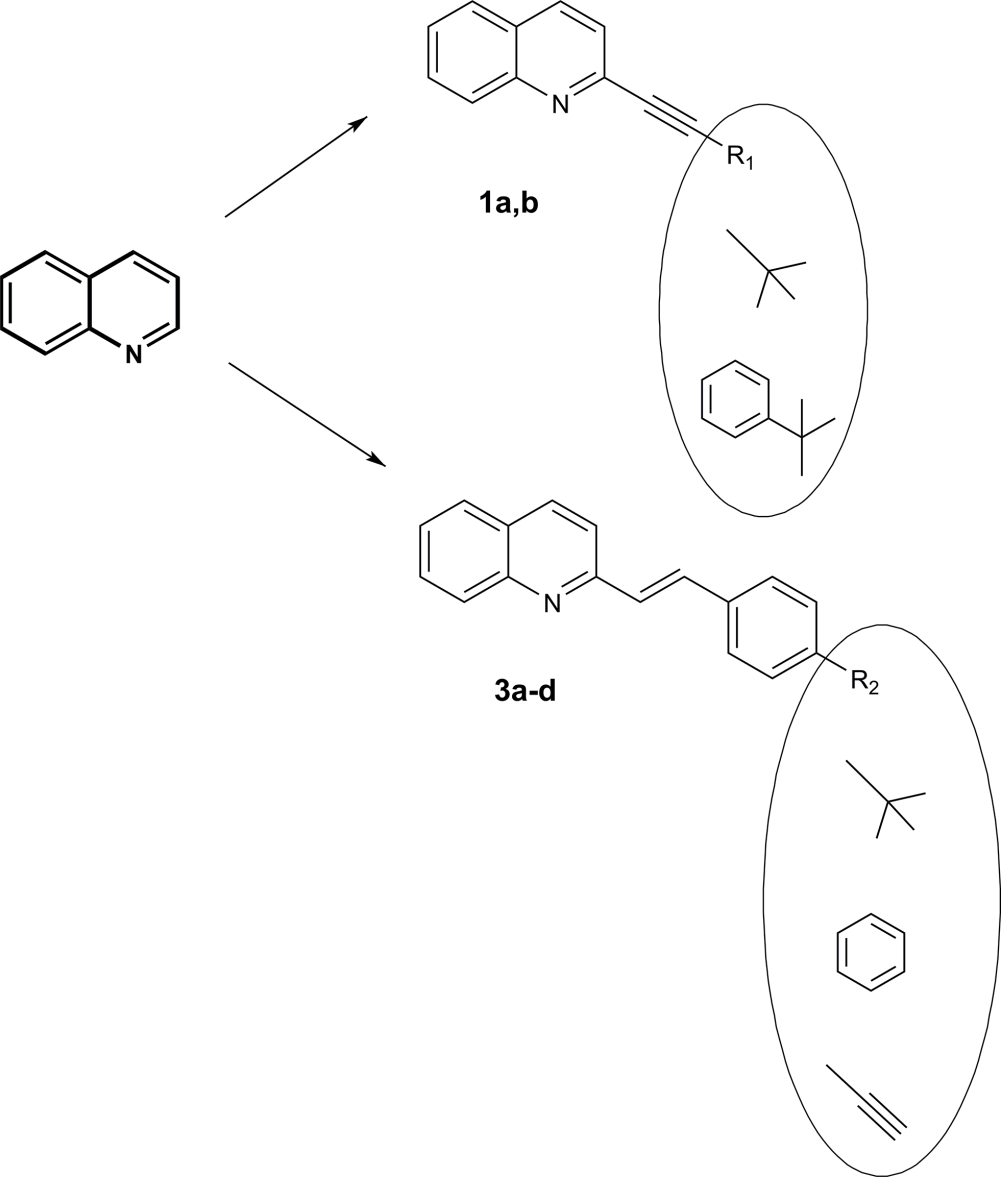
Structures of the quinoline derivatives.

In our group, quinoline derivatives are being broadly investigated for their chemistry [9,10] and possible applications [11–13]. These researches have resulted in some highly active anticancer [14,15] and antifungal [16] agents. In the present study, we describe the structural, electronic and optical properties of novel styrylquinolines and their simple analogs. The concept of the design of quinoline dyes is depicted in **Fig 1**. It combines the alkyne analogs of **1a-b** and styrylquinolines with an elongated lipophilic chain **3a-d**.

Such structural features are necessary for good fluorescent properties. On the other hand, the compounds do not have the functional groups that are required for their biological activity, e.g., the hydroxyl group at the C-8 position in a quinoline ring. This may suggest their low cytotoxicity and relative indifference to biological systems. With this in mind, we applied steady state UV-VIS absorption and emission spectroscopy in order to characterize their photophysical behavior. We applied *ab initio* molecular computing using the density functional theory (DFT) and time-dependent DFT (TD-DFT) calculations to gain insight into the electronic structure of the molecules that were being considered and in order to fully understand the experimental data. The impact of the structural features on the emission efficiency of styrylquinoline dyes is particularly important in terms of the development of novel fluorophores that might be suitable for various labeling applications.

## Materials and Methods

### A. General experimental method

All of the reagents were purchased from Aldrich. A Kieselgel 60, 0.040–0.063 mm (Merck, Darmstadt, Germany) was used for column chromatography. The TLC experiments were performed on alumina-backed silica gel 40 F254 plates (Merck, Darmstadt, Germany). The plates were illuminated under UV (254 nm). The melting points were determined on an Optimelt MPA-100 apparatus (SRS, Stanford CA). The purity of the final compounds was checked using HPLC. Detection wavelengths of 210 and 250 nm were chosen for detection. The purity of individual compounds was determined from the area peaks in the chromatogram of the sample solution in order to ensure >95% purity. UV spectra (λ, nm) were determined on a Waters 2996 Photodiode Array Detector (Waters Corp., Milford, MA, U.S.A.) in a methanolic solution (ca. 6×10^-4^mol) and log ε was calculated for the absolute maximum λ_max_ of individual target compounds. All NMR spectra were recorded on a Bruker AM-series, Bruker BioSpin Corp., Germany. The working frequency is given for each compound. Chemical shifts are reported in ppm (δ) against the internal standard, Si(CH_3_)_4_. Easily exchangeable signals were omitted when diffuse. Signals are designated as follows: s, singlet; d, doublet; dd, doublet of doublets; t, triplet; m, multiplet; bs, broad singlet.

Compounds **1a**, **1b**, and **3a** were synthesized according to the literature [17].

#### Preparation of nanosized palladium particles on the copper (5% Pd/Cu)

A solution of methanol (1050 mL) and aqueous ammonia (25 wt. %, 370 mL) was stirred for 15 min, followed by the addition of tetraethyl orthosilicate (75.09 mL). The reaction mixture was vigorously stirred for 2 h at room temperature to produce the colloidal silica suspension. The resultant colloidal silica suspension was centrifuged. Deionized water (100 mL) was added to the colloidal silica and the mixture was placed in an ultrasound bath and sonicated for 20 min. Then, an aqueous saturated solution containing 1.72g PdCl_2_ was added dropwise into the suspension of colloidal silica and mixed in an ultrasound bath for 20 min. The mixture was dried at 60–90°C for 12 h, ground and sieved. Finally, the reduction was conducted in an oven under hydrogen at 500°C for 1 h to produce nanosized metallic particles that were dispersed on the SiO_2_ intermediate carrier. Nanosized palladium particles were dispersed on the SiO_2_ (0.55 g) and Cu (0.5 g) was added to the deionized water (100 mL) and stirred for 10 min. Then, a freshly prepared sodium hydroxide solution (80 mL 40% w/w) was added and the suspension was stirred for 1 h. Next, the suspension was cooled to room temperature and allowed to form sediment. After decantation, the sediment was washed to neutral pH with deionized water. The resulting preparation of the catalyst was dried to a constant mass at room temperature [18].

**General method** synthesis of substrates **2b** and **2c** In a tightly sealed tube (septa system), aryl halides (5.5 mmol) and 5% nanocatalyst Pd/Cu, PPh_3_ (17 mg) were suspended in dry triethylamine (10 mL). The mixture was placed in an ultrasound bath and sonicated for 5 min. Then, the acetylene compound (5.6 mmol) was added and the mixture was stirred for 3 h. The mixture was cooled to room temperature and the catalyst was centrifuged, filtered and washed with ethyl acetate (3 x 10 mL). The filtrate was washed three times with deionized water (3 x 15 mL) and then dried over magnesium sulfate, filtered and concentrated under reduced pressure to give the product.

*Synthesis of 4-[(trimethylsilyl)ethynyl)benzaldehyde* (**2b**): The product was obtained in a quantitative yield, melting point 62–63°C. ^1^H NMR (400 MHz, DMSO-*d*6) *δ*: 10.02 (s, 1H), 7.90 (d, *J* = 8.55 Hz, 2H), 7.67 (d, *J* = 8.10 Hz, 2H), 0.26 (s, 9H). ^13^C NMR (101 MHz, DMSO-*d*6) *δ*: 192.34, 136.20, 132.74, 130.04, 127.83, 104.57, 99.21, 0.18.

*Synthesis of 4-(phenylethynyl)benzaldehyde*(**2c**): The product was obtained in a quantitative yield, melting point 95–98°C. ^1^H NMR (400 MHz, CDCl_3_): *δ* 10.40 (s, 1H), 7.89 (d, *J* = 8.2 Hz, 2H), 7.70 (d, *J* = 8.10 Hz, 2H), 7.58 (m, 2H), 7.40 (m, 3H). ^13^C NMR (101 MHz, CDCl_3_) *δ* 191.41, 135.44, 132.12, 131.81, 129.59, 128.98, 128.49, 122.52, 93.47, 88.53.

#### 1. Compound 3b

0.4 mL quinaldine (3 mmol) was dissolved in 10 mL of acetic anhydride with 3 mmol of aldehyde **2b.** The resulting mixture was stirred under argon at 130°C for 20 h. After evaporation to dryness, the product was purified by a short SiO_2_ column (eluent–ethyl acetate/hexane). The solution was then cooled and concentrated under reduced pressure. Next, the mixture was washed with diethyl ether. The product was obtained as a yellow solid with yields of 55%, melting point 191°C. ^1^H NMR (400 MHz, DMSO-d6) δ: 8.15 (d, J = 8.6 Hz, 1H), 8.12 (d, J = 8.5 Hz, 1H), 7.81 (d, J = 8.1 Hz, 1H), 7.77–7.71 (m, 1H), 7.67 (d, J = 8.7 Hz, 2H), 7.59 (d, J = 8.3 Hz, 2H), 7.55–7.50 (m, 3H), 7.43 (d, J = 16.3 Hz, 1H), 0.30 (s, 9H). ^13^C NMR (101 MHz, DMSO-d6) δ: 155.80, 148.17, 137.34, 137.00, 33.53, 132.64, 130.56, 130.32, 129.22, 128.26, 128.06, 127.89, 127.60, 126.78, 122.59, 120.60, 105.69, 96.08, 0.28, MS (EI) m/z: [M+H]^+^ Calcd for C_22_H_21_NSi 328.16; Found 328.35.

#### 2. Compound 3c

K_2_CO_3_ was added to **3b** dissolved in MeOH and the resulting mixture was stirred for 2 h at 30°C. The mixture was concentrated and added to water/diethyl ether and further extracted with diethyl ether. The organic layers were washed with water and brine and dried over MgSO_4_. After the evaporation of the solvent, a yellow solid was obtained with a yield of 90%, melting point 154°C. ^1^H NMR (400 MHz, DMSO-d6) δ: 8,16 (d, J = 8.6 Hz, 1H), 8.11 (d, J = 8.5 Hz, 1H), 7.81 (d, J = 7.9 Hz, 1H), 7.76–7.71 (m, 1H), 7.70–7.65 (m, 2H), 7.62 (d, J = 8.3 Hz, 2H), 7.56–7.51 (m, 3H), 7.44 (d, J = 16.3 Hz, 1H), 3.19 (s, 1H), ^13^C NMR (101 MHz, DMSO-d6) δ: 155.58, 148.26, 137.00, 136.48, 133.47, 132.56, 130.04, 129.86, 129.25, 127.49, 127.44, 127.11, 126.36, 122.13, 119.43, 83.60, 78.39, MS (EI) m/z: [M+H]^+^ Calcd for C_19_H_13_N 256.12; Found 256.28. Anal. Calcd for C_19_H_13_N: C, 89.38; H, 5.13; N, 5.49. Found: C, 89.64; H, 5.47; N, 5.32.

#### 3. Compound 3d

0.4 mL quinaldine (3 mmol) was dissolved in 10 mL of acetic anhydride with 3 mmol of aldehyde **2c**. The resulting mixture was stirred under argon at 130°C for 20 h. After evaporation to dryness, the product was purified by a short SiO_2_ column (eluent–dichloromethane). The solution was concentrated under reduced pressure. Next the mixture was washed with diethyl ether. The product was obtained as a pale yellow solid with yields 50%, melting point 201–203°C. ^1^H NMR (400 MHz, CDCl_3_) δ: 8.15 (d, J = 8.6 Hz, 1H), 8.12 (d, J = 8.5 Hz, 1H), 7.81 (d, J = 8.1 Hz, 1H), 7.77–7.71 (m, 1H), 7.67 (dd, J = 13.7, 8.4 Hz, 4H), 7.61–7.56 (m, 4H), 7.53 (t, J = 7.5 Hz, 1H), 7.45 (d, J = 16.3 Hz, 1H), 7.41–7.35 (m, 3H). ^13^C NMR (101 MHz, CDCl_3_) δ: 155.70, 148.29, 136.44. 133.65, 132.03, 131.65, 129.83, 129.74, 129.26, 128.39, 127.53, 127.43, 127.20, 126.31, 123.39, 123.23, 119.42, 90.84, 89.46, MS (EI) m/z: [M+H]^+^ Calcd for C_25_H_17_N 332.14; Found 332.33. Anal. Calcd for C_25_H_17_N: C, 90.60; H, 5.17; N, 4.23. Found: C, 90.35; H, 5.11; N, 4.40.

### B. Cell culture

Human colon adenocarcinoma cells (HCT116) and normal human fibroblast (GM 07492) cells were obtained from the American Type Culture Collection. Cells were grown as monolayer cultures in 75 cm^3^ flasks (Nunc) in Dulbecco's Modified Eagle Medium (DMEM). The medium was supplemented with 12% heat inactivated fetal bovine serum (PAA) and for GM 07492 with 15% fetal bovine serum (Gibco) and 100 μg/mL of gentamycin (Gibco). The cell lines were maintained at 37°C in a 5% CO_2_ incubator and passaged every 3–4 days as required.

### C. Cytotoxicity assay

Exponentially growing cells were harvested through the trypsinization of sub-confluent cultures. Cells were seeded into 96-well cell culture microtiter plates (Nunc) at concentrations of 5.0 x 10^3^ cells per well and cultured for 24 h. After this time, the growth medium was exchanged for a medium containing the compounds in concentrations ranging from 0.5 μM-25 μM. Stock solutions of the compounds being investigated were prepared in sterile DMSO. The final concentration of DMSO in the medium did not exceed 0.2%. After a 72 h incubation with the compounds being investigated under standard cell culture conditions, the medium was replaced with 100 μL of DMEM without phenol red. The metabolic activity of viable cells was determined by adding 20 μL of CellTiter 96®AQueousOne Solutions–MTS (Promega) to each well followed by a 1 h incubation. The MTS assay is a colorimetric method for determining the number of viable cells. A standard solution containing 100 μL of DMEM without phenol red and 20 μL of MTS solution was used to determine”blank” absorbance. The absorbance was measured at 490 nm using a SynergyTM4 microplate reader (BioTek). The inhibitory concentration (IC_50_) was defined as the compound concentration that was necessary to reduce the proliferation of cells to 50% of the untreated control cells and expressed as means ± standard deviation (SD) in GraphPad Prism 5 software. Each individual compound was tested in triplicate in a single experiment with each experiment being repeated 3–5 times.

### D. The cellular staining

To visualize the accumulation of the compounds within the cells, 10×10^3^ HCT116 cells in a 300 μL growth medium were plated into an 8-well LabTek chambered cover glass (Nunc) and incubated under standard conditions at 37°C in a humidified atmosphere at 5% CO_2_ for 24h. After this, the cells were treated with the compounds being tested (**3a**, **3c** and **3d**) at a concentration of 25 μM and incubated for a further 2h. After incubation the cells were washed three times with PBS and then 300 μL of PBS was added. Observation of the cells was carried out using an inverted fluorescence microscope (IX81, Olympus) immediately after staining.

### E. Spectroscopic studies

The absorption and fluorescence spectra were measured at room temperature in a 10 mm quartz cell with a U-2900 spectrophotometer (Hitachi) and an F-7000 spectrofluorimeter (Hitachi), respectively. The styrylquinoline stock solutions (10mM) that had been prepared in DMSO were further diluted with appropriate spectroscopic grade solvents to working concentrations starting from0.1mM (1% DMSO).The fluorescence quantum yields were measured using the comparative method with anthracene in cyclohexane as a reference (*ϕ*_*ref*_ = 0.34) [19]. We prepared standard and test solutions of decreasing concentrations in order to provide absorbance in the range of 0.1 to 0.02 at the excitation wavelength. The fluorescence spectra of all of the solutions were measured at room temperature in a 10 mm cell using an F-7000 spectrofluorimeter (Hitachi). The fluorescence quantum yields of the dyes that were tested were calculated as follows: $\phi_{dye}=\phi_{ref}\cdot \left(\alpha_{dye}/\alpha_{ref}\right)\cdot \left(\eta_{dye}^{2}/\eta_{ref}^{2}\right)$, where subscripts *dye* and *ref* indicate test and standard samples, respectively, *ϕ* is the fluorescence quantum yield, *α* is the gradient obtained from the plot integrated fluorescence intensity versus absorbance and *η* is the refractive index of solvents that were applied.

### F. Calculations

DFT and TD-DFT calculations for styrylquinoline derivatives were performed using the Gaussian 09 software package [20] with the B3LYP exchange-correlation functional [21] and 6–31+G(d,p) basis set. The effects of the solvent were evaluated using the PCM model[22–24] in which a cavity is created *via* a series of overlapping spheres [25] with standard dielectric constants (ε) of 46.826 for DMSO. Electronic absorption transition energies for 32 spin-allowed singlet-singlet (S_0_→S_n_) and fluorescence emission spectra made of the 8 spin-allowed S_n_→S_0_ transitions were considered using the external iteration (EI) approach [26–29]. The molecular electron densities for each derivative were determined from the wave functions using the CUBE option implemented in Gaussian 09 and visualized using GaussView 5.0. The molecular energy levels were analyzed using Chemissian software.

## Results and Discussion

### A. Synthesis and characterization

**Fig 2:**
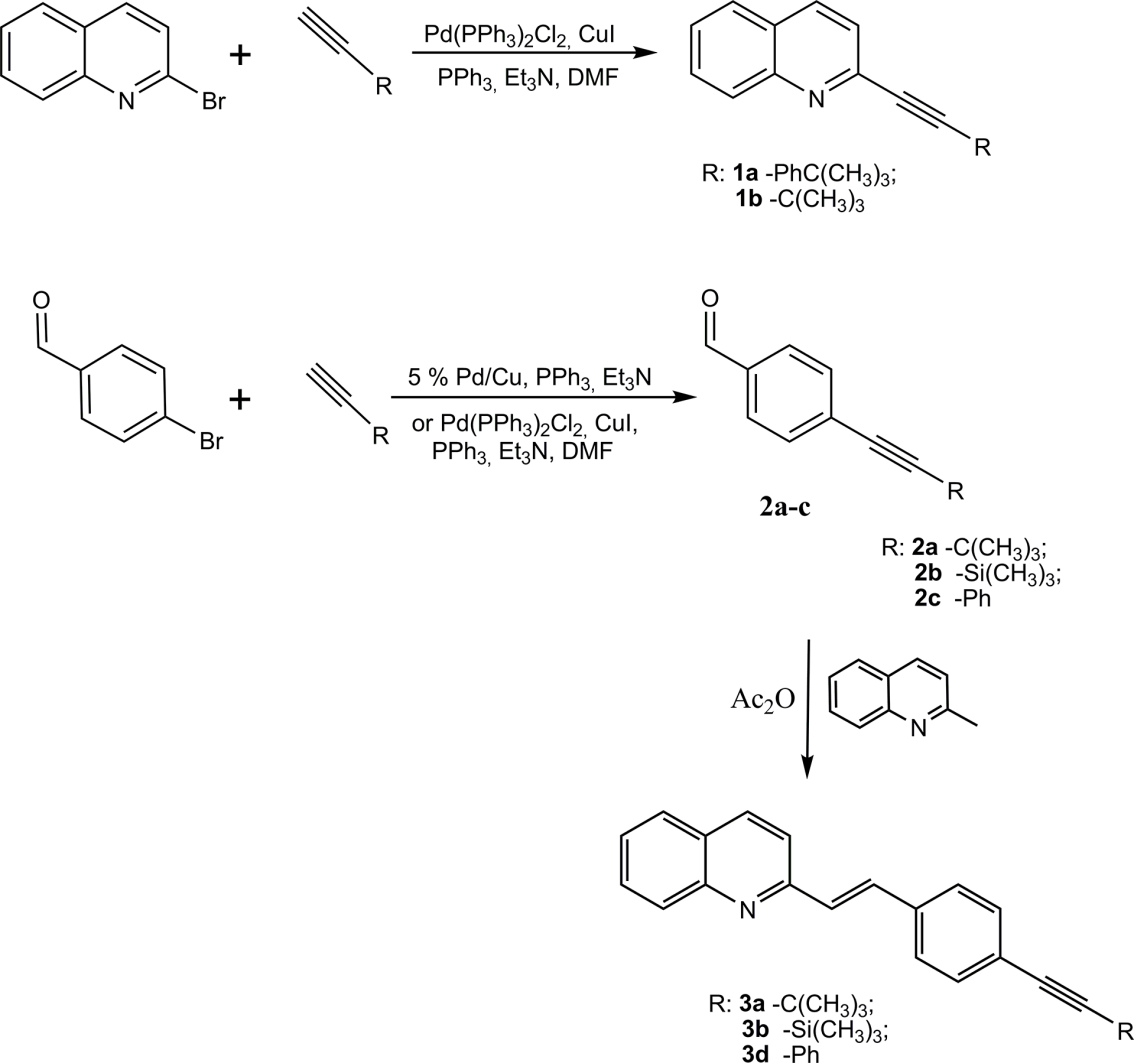
Sonogashira cross-coupling reaction and synthesis of SQLs. The direct alkylation of the 2-bromoquinoline produced analogs of styrylquinolines**1a-b** [17].

Our synthetic approach is depicted in **Fig 2**. The compounds that were investigated were prepared using the Sonogashira reaction in homogeneous or heterogeneous conditions.

**Fig 3:**
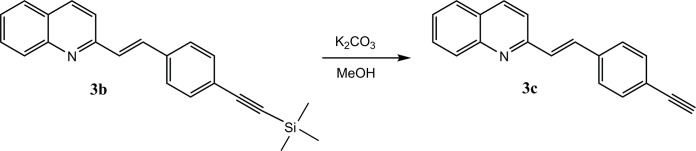
Hydrolysis of compound 3b.

Compounds **3a**, **3b**, **3d** were obtained according to the standard method [9, 10, 30] from quinaldine and aldehydes **2a-c** in acetic anhydride, while aldehydes **2b-c** were obtained in the Sonogashira coupling in heterogeneous conditions on a nano-Pd catalyst. For the preparation of the bimetallic catalyst 5% Pd/Cu, we modified a previously described method [18].The overall activity of the nano-particle catalysts depends mainly on the effectiveness of the distribution of the metallic grains in the matrix [31]. The bimetallic nano-Pd/Cu was obtained by digesting the carrier in nano-Pd/silica and transferring the nanoparticles on to the electrolytic copper. We found that digestion of the material dispersed in an excess of the NaOH resulted in a shortened time of the procedure and reduced the temperature. Such prepared nanoparticles of the catalyst retain their high activity[18]. This catalytic system provided us with quantitative conversions. Compound **3c** was obtained through the hydrolysis of **3b** immediately after its synthesis as is shown in **Fig 3**. All of the compounds that were obtained were characterized using NMR and mass spectroscopy.

### B. Biological activity

The antiproliferative activity of the compounds that were synthesized was assessed using the MTS assay. The results from the cytotoxicity assay are shown in **Table 1**. In general, the compounds that were tested appeared to be inactive against the HCT116 cell lines.

**Table 1:** Antiproliferative activity.

com.	IC_50_ [μM]	
	HCT116	GM 07492
**1a**	>25^*a*^	>25
**1b**	>25	>25
**3a**	21.18±3.36	>25
**3c**	>25	>25
**3d**	>25	>25

^*a*^Results are expressed as mean ± standard deviation from 3–5 experiments.

All of the tested compounds were also examined for their cytotoxic effects against the normal human fibroblasts. All of the compounds that were tested proved to be inactive. In the context of the potential use of these compounds as fluorescent dyes, the lack of cytotoxicity is a significant advantage.

### C. Cellular imaging

**Fig 4:**
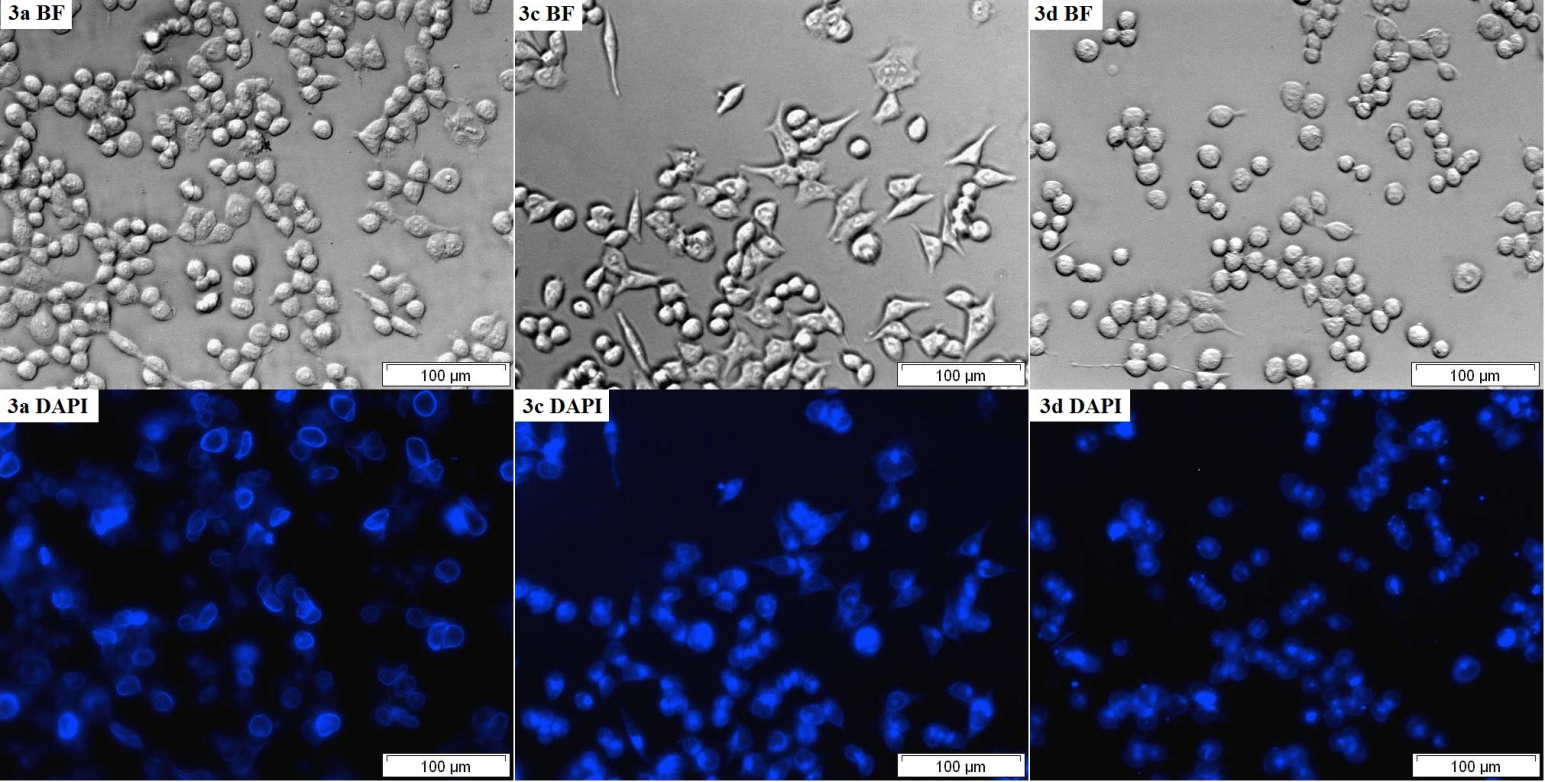
Live cell imaging of the HCT116 cells following treatment with 3a, 3c and 3d using bright field optical microscopy (BF) and fluorescence microscopy (DAPI filter).

The excitation waves for compounds that were tested are relatively short, which can cause problems in biological applications. The sample autofluorescence and poor background suppression are the most important here. Nevertheless, we decided to perform the appropriate experiments and got some interesting results. For the staining experiments, we selected the most effective compounds **3a**, **3c** and **3d**. The cellular staining of selected compounds in the human colon carcinoma cells (HCT116) is presented in **Fig 4**. After a 2h incubation, all of the compounds efficiently penetrated the cellular membranes and a strong blue signal was observed. Interestingly, **3a** (*t*-butyl derivative) seems to locate primarily in plasma membrane rather than in the internal organelles. On the other hand, compounds **3c** (R = H) and **3d** (R = Ph) accumulated intracellularly. The micrographs of these two compounds are comparable to lysosome staining. The localization of the **3c** and **3d** may be partially explained based on the physicochemical properties of the tested compounds. Styrylquinolines are weak bases and their lipophilicity is relatively high (AlogP: **3a**: 6.31, **3c**: 4.73, **3d**: 6.51). Such compounds generally exhibit lysosomotropism. The predominant localization of the **3a** in plasma membrane remains unclear.

### D. Fluorescent properties

The absorption and emission properties of the compounds that were tested were measured in several solvents that had decreasing polarity (DMSO > ethanol > chloroform). The absorption spectra that were registered did not show any evident solvent-dependent spectral shifts (see **Table 2**). In general, the spectrum of **1b** was the one that shifted most to shorter wavelengths. All of the compounds have relatively high values of molar absorption coefficients (ε) (see **Table 2**). Interestingly, we observed that the ε values were significantly higher in DMSO than in other solvents. The highest ε value was observed for **3d** in DMSO (ε = 44 000 M^-1^cm^-1^ at 360 nm).

**Table 2:** Fluorescent and absorption properties of quinolone dye solutions in various solvents.

	λ_max_ [nm](ε·10^3^ [M^-1^ cm^-1^])	λ_em_ [nm]	Stokes shift [nm]	*ϕ*_*dye*_
DMSO				
**1a** (wk39)	278 (17.9)	367	23	0.01
**1b** (wk40)	288 (0.51)	**-**	**-**	-
**3a** (wk44)	260 (37.0)	407	47	0.06
**3c** (mk7)	342 (33.8)	408	52	0.01
**3d** (mk9)	360 (44.0)	411	51	0.32
Ethanol				
**1a** (wk39)	279 (14.8)	369	23	0.19
**1b** (wk40)	333(5.91)	350	17	0.01
**3a** (wk44)	347 (18.4)	399	40	0.06
**3c** (mk7)	344 (18.7)	390	32	0.04
**3d** (mk9)	356 (24.5)	412	56	0.24
Chloroform				
**1a** (wk39)	280 (14.7)	361	15	0.11
**1b** (wk40)	332 (6.58)	-	-	-
**3a** (wk44)	347 (18.4)	400	38	0.06
**3c** (mk7)	344 (18.5)	389	30	0.03
**3d** (mk9)	357 (24.1)	410	50	0.38

**Fig 5:**
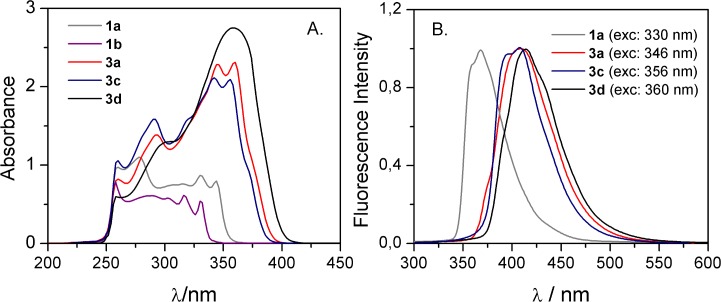
UV-VIS absorption (A) and normalized fluorescence (B) spectra of styrylquinolines solutions in DMSO.

The emission spectra of the compounds that were synthesized covered the colors from violet to blue. For **1a** in the DMSO solution, the maximum fluorescence emission was observed in the ultraviolet region (λ_em_ = 367 nm) while for the others the maximum was blue-shifted and was observed in the visible region of the electromagnetic spectrum (λ_em_ = 407–411 nm, see **Fig 5-B**). For solvents that had a lower polarity, the emission maxima were slightly shifted towards shorter wavelengths. In the case of **1b** the weak fluorescence emission was observed only for solutions prepared in ethanol.

The Stokes shift reflects a loss of energy between the excitation and emission that was observed in the solution. As a result, the emission spectrum is naturally shifted toward longer wavelengths. A small value of the Stokes shift can negatively affect the sensitivity of fluorescence detection. The differences that were observed between excitation and emitted light for the dyes described in this report are applicable (15–56 nm, see **Table 2**).

**Fig 6:**
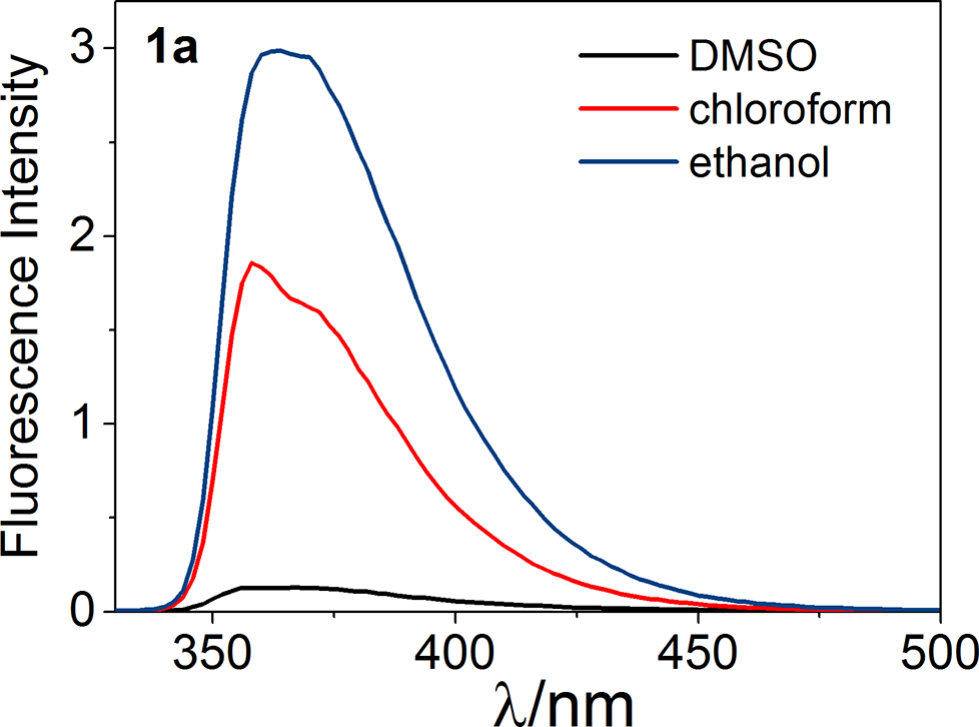
The comparison of emission spectra of 1a in solvents of various polarity

The fluorescence quantum yield is one of the key parameters of fluorophores and permits a quantitative description of the fluorescence phenomenon. It is defined as the number of photons being emitted relative to the number of photons being absorbed. The quantum yields that were calculated are presented in **Table 2**. The strong influence of solvent on fluorescence emission was observed for **1a** (**Fig 6**). Changing the solvent from DMSO to ethanol and chloroform led to a noticeable growth of fluorescence efficiency from 0.01 to 0.19 and 0.11, respectively. Among the compounds that were investigated, **3d** had a significantly higher quantum efficiency than the others (e.g., 0.38 in chloroform), which means that incorporating an additional aromatic unit positively affects the fluorescent properties of a fluorophore. The same seems to be true for compounds **1a** and **1b** for which only a phenyl substituted structure revealed some fluorescent potency. This is in agreement with the general opinion about the nature of the fluorophoric properties of the aromatic core. Polycyclic systems and scaffolds packed with π conjugated bonds are the base of the most effective fluorophores. Recently, Yamaguchi and coworkers presented an apparent correlation between the structure of π conjugated hydrocarbons and their quantum yield of fluorescence [32]. According to that report, both the fluorescence emission maximum and quantum yield are higher in longer π conjugation in an S_1_ state. Namely, the longer the linear donor/acceptor conjugated dipole the better its fluorescent properties. The compounds that were tested, although unsubstituted on account of their biological indifference, should follow this rule to some extent. To evaluate this, we performed some additional calculations as follows.

### E. Theoretical calculations

To better understand and characterize the absorption spectra of the compounds that were tested, TD-DFT calculations were performed with B3LYP exchange-correlation functional and 6–31+G(d,p) basis set.

The most important parameters such as transition energies, oscillator strengths (f) and the main configurations are listed in **Table 3**along with experimental absorption data. The rest of the electronic transitions were omitted due to the very low intensity of parameter f and were not taken into account during further data analysis.

**Table 3:** Electronic transition data obtained by EI TD-DFT/B3LYP/6-31+G(d,p) using a PCM model (solvent–DMSO) for quinoline dyes at the DFT optimized geometry.

com.	Electronic transitions	Theoretical λ_max_[nm]	f	molecular orbital(MO)	% coefficient	experimental λ_max_[nm]
**1a**	S_0_→S_1_	335.9	0.997	HOMO→LUMO	68	344
	S_0_→S_2_	318.3	0.012	HOMO_-1_→LUMO	62	330
				HOMO→LUMO_+1_	27	
	S_0_→S_4_	281.2	0.585	HOMO_-1_→LUMO	28	278
				HOMO→LUMO_+1_	60	
**1b**	-	-	-	-	-	330
	-	-	-	-	-	318
	S_0_→S_3_	310.6	0.193	HOMO→LUMO	62	304
				HOMO→LUMO_+1_	20	
	S_0_→S_4_	293.3	0.021	HOMO_-1_→LUMO	57	288
				HOMO→LUMO	27	
				HOMO→LUMO_+1_	28	
**3a**	S_0_→S_1_	381.4	1.041	HOMO→LUMO	69	360
				HOMO_-1_→LUMO	13	
	S_0_→S_2_	357.1	0.364	HOMO_-1_→LUMO	67	346
				HOMO→LUMO	11	
	S_0_→S_6_	292.4	0.253	HOMO_-3_→LUMO	41	292
				HOMO→LUMO_+1_	25	
				HOMO→LUMO_+2_	39	
**3c**	S_0_→S_1_	377.8	0.949	HOMO→LUMO	70	356
	S_0_→S_2_	329.6	0.063	HOMO_-1_→LUMO	55	342
				HOMO→LUMO_+1_	39	
	S_0_→S_4_	295.9	0.504	HOMO_-1_→LUMO	41	290
**3d**	S_0_→S_1_	401.9	1.632	HOMO→LUMO	70	360
	S_0_→S_3_	309.5	0.298	HOMO_-1_→LUMO	28	305

The applied theoretical approach correctly predicted the experimental maximum absorption data for **1a, 3a, 3c** and **3d**. On the other hand, the experimental bands for **1b** were not fully reflected by theory. This could be due to the geometry of the molecule. In general, these calculations confirmed that the number of double and triple bonds shifts the absorption maximum to longer wavelengths (i.e. bathochromic shift). This observation was further confirmed with the HOMO/LUMO orbitals and energy gap (ΔE_H-L_) analysis according to the scheme presented in the literature [33, 34]. The theoretical and experimental data are summarized in **Table 4**. It has been found that due to the growing energy gap (ΔE_H-L_), the level of conjugation decreases in the following order **3d**→**3a**→**3c**→**1a**→**1b**. In conjugated molecules, the energy gap between HOMO and LUMO orbitals is lower, which increases the possibilities of electron promotion at longer waves. This is reflected in our results as the experimental absorption wavelengths that were observed for **3d** and **3a** are more red-shifted with respect to the least-coupled system – **1b**.

**Table 4:** Negative orbital (au) of the HOMO (-E_HOMO_) and LUMO energies (-E_LUMO_), HOMO–LUMO band gap energies that were calculated by PCM//DFT-DFT/B3LYP/6-31+G(d,p) using the PCM model (solvent–DMSO) and the theoretical excited energies (E_g_) for dyes.

com.	-E_HOMO1_	-E_HOMO_	-E_LUMO_	-E_LUMO+1_	ΔE_H-L_[eV] (DFT)	E_g_[eV] (TD-DFT)
**1a**	6.77	6.52	2.16	1.39	4.36	3.39
**1b**	6.86	6.55	2.04	1.15	4.51	3.96
**3a**	6.65	5.88	2.38	1.40	3.50	3.25
**3c**	6.62	6.49	2.29	1.44	4.19	3.28
**3d**	6.59	5.82	2.48	1.62	3.33	3.08

According to the theoretical data, the experimental absorption maxima for **3c** and **3d** are characterized by S_0_→S_1_ (assigned to π→π*) transitions that are composed of HOMO→LUMO configurations (see **Table 3**). The presence of a *tert*-butyl group in molecule **3a** prevents π-conjugation, and consequently, the S_0_→S_1_ electronic transition is a mixture of HOMO→LUMO (69%) and HOMO_-1_→LUMO (13%) configurations.

**Fig 7:**
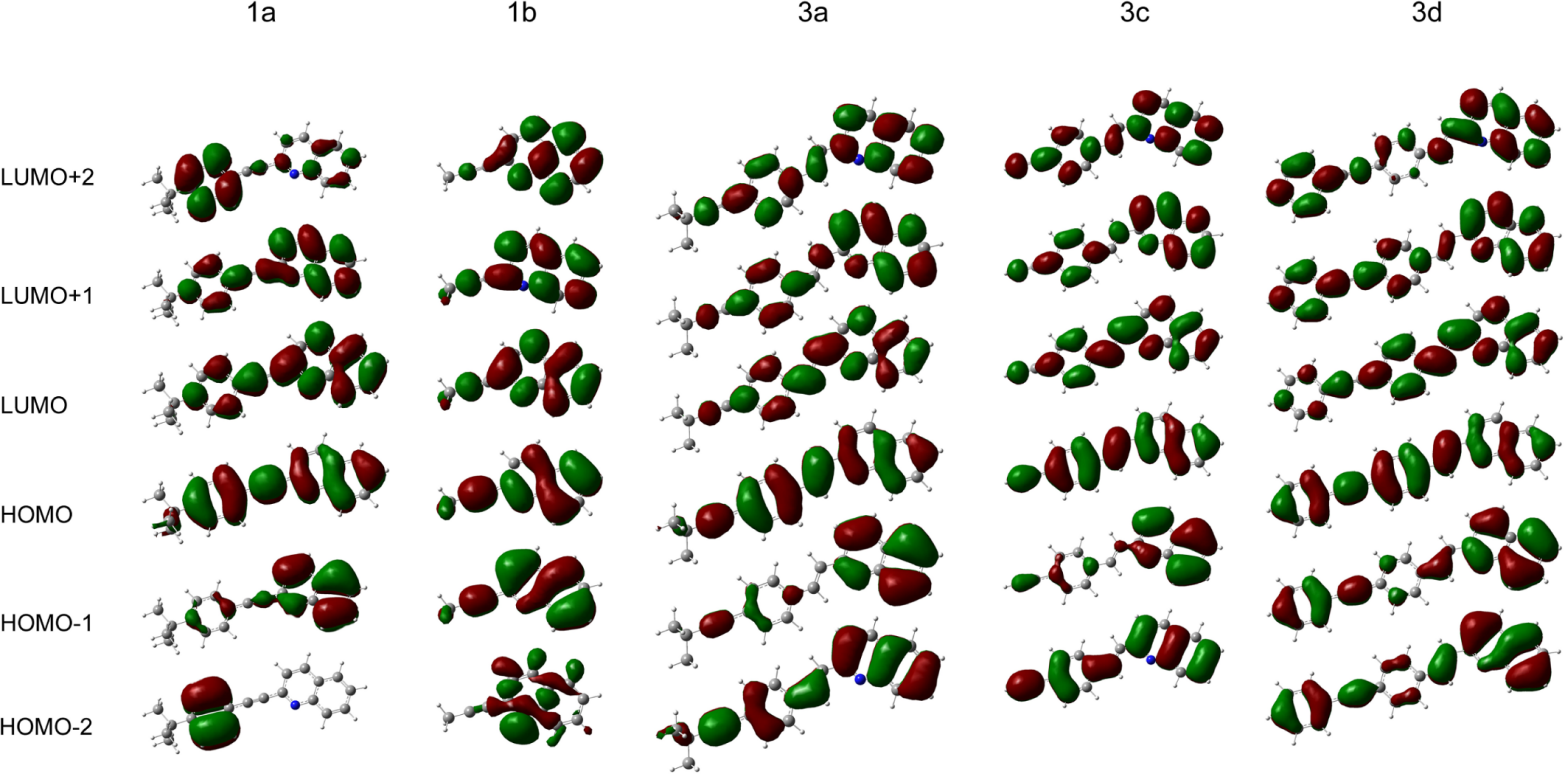
Molecular orbitals of the five quinoline derivatives obtained at the TD-B3LYP/6-31+G(d,p) theory level.

In the case of **1b** the low intensity experimental bands corresponded with the theoretical bands due to the S_0_→S_3_ and S_0_→S_4_ electronic transitions, while the most intensive experimental bands (not reflected by theory) would be associated with S_0_→S_1_ and S_0_→S_2_. It is noteworthy that the isosurfaces of the HOMO and LUMO orbitals are localized through the central double bond and the double bonds of the rings in all of the molecules (**Fig 7**), while LUMO distribution is also localized on the nitrogen.

The higher electronic S_0_→S_n_ (n = 2, 3, 4) (π→π*) transitions for almost all of the systems generally consisted of mixed HOMO_-1_→LUMO and HOMO→LUMO_+1_. Another character is observed for **3a** where the second-lying S_0_→S_2_ transition is due to two molecular orbital contributions (HOMO_-1_→LUMO + HOMO→LUMO) and the lowest-lying S_0_→S_6_ transition corresponded to the HOMO_-3_→LUMO + HOMO→LUMO_+1_ + HOMO→LUMO_+2_ excitations. Moreover, the atomic orbitals of the carbon of the double bond provide a significant contribution to HOMO but no contribution to HOMO_-1._ In the case of LUMO_+1_, the electron distribution was concentrated on the atoms of the pyridine and phenyl ring. Noticeably, electronic S_0_→S_n_ (n = 3, 4) (π→π*) transitions for **1b** consisted of mixed HOMO_-1_→LUMO and HOMO→LUMO_+1_ as well as HOMO–LUMO molecular orbitals.

The molecular orbital analysis confirmed that the difference between styrylquinoline properties depends on the conjugation. The orbital energy levels increase for HOMO (**1b**→**1a**→**3c**→**3a**→**3d**) and LUMO (**3d**→**3a**→**3c**→**1a**→**1b**) with respect to the number of conjugated groups. This relationship is clear from the point of view of the electronic properties of the molecular fragments with alkyne group (C≡C–H; **3c**), which are the most electron-withdrawing. On the other hand, the strong electron-donating and activating phenyl group that was attached to the C≡C fragment of **3d** molecule makes this compound the most nucleophilic of all those studied.

The fluorescence emission energies for the spin-allowed singlet-singlet (S_n_→S_0_) transitions were calculated for the dye solutions in DMSO using the external iteration (EI) TD-DFT//B3LYP/6-31+G(d,p) approach. The transition energies, oscillator strengths and main configurations that were computed for the most relevant excited states of each molecule were compared with the experimental values and are summarized in **Table 5**.

**Table 5:** Emission data obtained by EI TD-DFT/B3LYP/6-31+G(d,p) using the PCM model (solvent–DMSO) for SQLs at the DFT optimized geometry.

com.	Electronic transitions	theoreticalλ_em_[nm]	f	Molecular orbital (MO)	% coefficient	ε_em_·10^3^	experimental λ_em_[nm]
**1a**	S_1_→S_0_	401.7	0.017	HOMO→LUMO	50	2	-
				HOMO_-1_→LUMO	48		
**1b**	S_1_→S_0_	362.7	0.835	HOMO→LUMO	70	37	367
	S_2_→S_0_	349.5	0.138	HOMO_-1_→LUMO	66	6	
				HOMO→LUMO_+1_	22		
**3a**	S_2_→S_0_	419.9	1.039	HOMO→LUMO	17	52	407
				HOMO_-2_→LUMO	69		
	S_3_→S_0_	385.9	0.485	HOMO→LUMO	17	24	
				HOMO_-2_→LUMO	66		
**3c**	S_1_→S_0_	421.1	1.487	HOMO→LUMO	70	48	402
**3d**	S_1_→S_0_	434.9	1.919	HOMO→LUMO	71	75	411

The wavelengths of the maximum emission that were calculated are in good agreement with the experimental values for all of the compounds. The theoretical emission value for **1b** was found to be very low despite the lack of the luminescence effect that was observed in the experiment. This may be the effect of the quenching process as the change of the solvent revealed some fluorescence (see **Table 2**). The theoretical data for **3a** explained the experimental emission peak as a mix of S_2_→S_0_ + S_3_→S_0_ electronic transitions within HOMO→LUMO and HOMO_-2_→LUMO (see **Table 5**).

**Fig 8:**
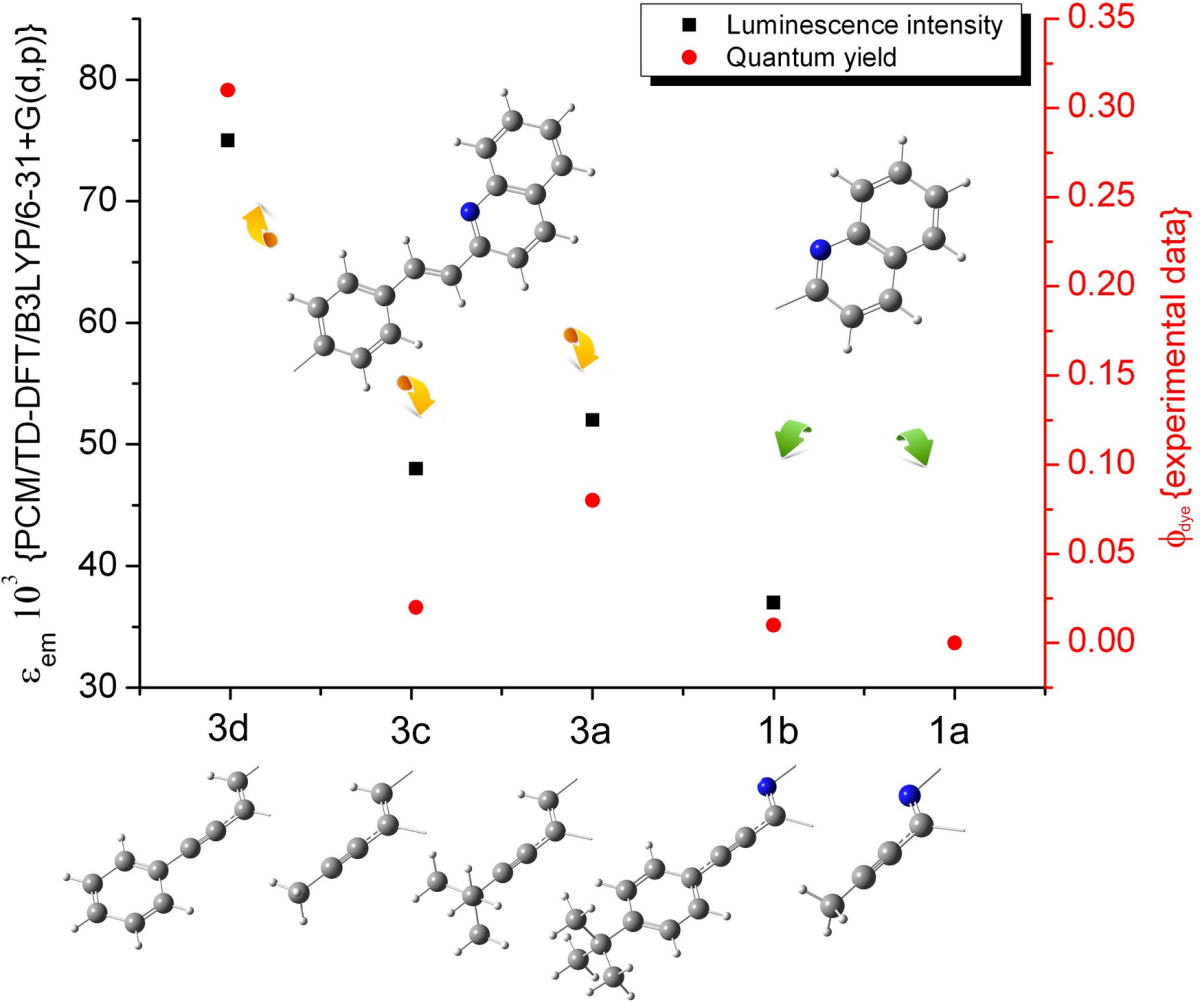
Theoretical extinction coefficient compared with the experimental quantum yield. Green arrows: a system with poor conjugation (**1a**, **1b**), yellow arrows: strongly conjugated molecules (**3a**, **3c**, **3d**).

The extinction coefficients that were obtained from the theoretical outcomes correspond with the experimental quantum yields (**Fig 8**). This correlation indicated that such a theoretical approach may be a simple and convenient tool for predicting the emission features of newly synthesized systems. As was expected, the more conjugated system had a higher emission intensity and quantum yield. Furthermore, the introduction of a strongly electron-donating group (e.g., benzene) in the alkyne linker–C≡C–significantly enhanced both of the features mentioned above.

## Conclusions

A series of five quinoline derivatives were designed based on the styrylquinoline system. These compounds were obtained according to the novel method of Sonogashira coupling on a bimetal nanocatalyst with a satisfactory to good yield. The preparation of the nanocatalyst was also improved according to known methods, which substantially improve the availability of this system. The compounds that were obtained were tested for their biological activity against human cancer cells and normal fibroblasts. Their absorption and fluorescent spectra were collected with quantum yields. DFT and TD-DFT calculations were carried out to confirm the experimental observations. The compounds that are presented have a low toxicity and are tolerable in biological systems in useful concentrations for a long time (72 h vs 2 h observation). Their fluorescent properties make them potentially interesting leading structures for further development. Moreover, the theoretical calculation that is presented allows the direction of the modification of basic compounds to intensify its luminescence parameters to be proposed.

## References

[1] Musiol R. Quinoline-based HIV integrase inhibitors. Curr Pharm Des. 2013;19: 835–849. 10.2174/1381612811319100008 23092281

[2] Musiol R, Serda M, Hensel-Bielowka S, Polanski J. Quinoline-based antifungals. Curr Med Chem. 2010;17: 1960–1973. 10.2174/092986710791163966 20377510

[3] Solomon R, Lee H. Quinoline as a privileged scaffold in cancer drug discovery. Curr Med Chem. 2011;18: 1488–1508. 10.2174/092986711795328382 21428893

[4] Cinar R, Nordmann J, Dirksen E, Müller TJJ. Domino synthesis of protochromic “ON-OFF-ON” luminescent 2-styryl quinolines. Org Biomol Chem. 2013;11: 2597–2604. 10.1039/c3ob27270b 23446649

[5] Nosova EV, Trashakhova TV, Ustyugov VS, Mochul’skaya NN, Valova MS, Lipunova GN, et al Fluorine-containing quinoline and quinoxalinestyryl derivatives: synthesis and photophysical properties. Russ Chem Bull. 2011;60: 942–947. 10.1007/s11172-011-0148-1

[6] Pillai S, Kozlov M, Marras SAE, Krasnoperov LN, Mustaev AA. New cross-linking quinoline and quinolone derivatives for sensitive fluorescent labeling. J Fluoresc. 2012;22: 1021–1032. 10.1007/s10895-012-1039-z 22450725 PMC3397220

[7] Rosania GR, Lee JW, Ding L, Yoon H-S, Chang Y-T. Combinatorial approach to organelle-targeted fluorescent library based on the styryl scaffold. J Am Chem Soc. 2003;125: 1130–1131. 10.1021/ja027587x 12553790

[8] Staderini M, Aulić S, Bartolini M, Tran HNA, González-Ruiz V, Pérez DI, et al A fluorescent styrylquinoline with combined therapeutic and diagnostic activities against Alzheimer’s and Prion diseases. ACS Med Chem Lett. 2013;4: 225–229. 10.1021/ml3003605 24900645 PMC4027132

[9] Machura B, Wolff M, Cieślik W, Musiol R. Novel oxorhenium(V) complexes of 8-hydroxyquinoline derivatives–Synthesis, spectroscopic characterization, X-ray crystal structures and DFT calculations. Polyhedron. 2013;51: 263–274. 10.1016/j.poly.2012.12.028

[10] Machura B, Wolff M, Kowalczyk W, Musiol R. Novel rhenium(V) complexes of 8-hydroxyquinoline derivatives–Synthesis, spectroscopic characterization, X-ray structure and DFT calculations. Polyhedron. 2012;33: 388–395. 10.1016/j.poly.2011.11.051

[11] Musiol R, Podeszwa B, Finster J, Niedbala H, Polanski J. An efficient microwave-assisted synthesis of structurally diverse styrylquinolines. Monatsh Chem. 2006;137: 1211–1217. 10.1007/s00706-006-0513-1

[12] Podeszwa B, Niedbala H, Polanski J, Musiol R, Tabak D, Finster J, et al Investigating the antiproliferative activity of quinoline-5,8-diones and styrylquinolinecarboxylic acids on tumor cell lines. Bioorg Med Chem Lett. 2007;17: 6138–6141. 10.1016/j.bmcl.2007.09.040 17904844

[13] Mrozek-Wilczkiewicz A, Kalinowski DS, Musiol R, Finster J, Szurko A, Serafin K, et al Investigating the anti-proliferative activity of styrylazanaphthalenes and azanaphthalenediones. Bioorg Med Chem. 2010;18: 2664–2671. 10.1016/j.bmc.2010.02.025 20303768

[14] Mrozek-Wilczkiewicz A, Serda M, Musiol R, Malecki G, Szurko A, Muchowicz A, et al Iron chelators in photodynamic therapy revisited: synergistic effect by novel highly active thiosemicarbazones. ACS Med Chem Lett. 2014;5: 336–339. 10.1021/ml400422a 24900837 PMC4027794

[15] Serda M, Kalinowski DS, Mrozek-Wilczkiewicz A, Musiol R, Szurko A, Ratuszna A, et al Synthesis and characterization of quinoline-based thiosemicarbazones and correlation of cellular iron-binding efficacy to anti-tumor efficacy. Bioorg Med Chem Lett. 2012;22: 5527–5531. 10.1016/j.bmcl.2012.07.030 22858101

[16] Musiol R, Jampilek J, Buchta V, Silva L, Niedbala H, Podeszwa B, Antifungal properties of new series of quinoline derivatives. Bioorg Med Chem. 2006;14: 3592–3598. 10.1016/j.bmc.2006.01.016 16458522

[17] Cieslik W, Musiol R, Korzec M. Synthesis of alkyne-substituted quinolines as analogues of allylamines. Int Bull Pharm Sci. 2012;1: 3–9.

[18] Korzec M, Bartczak P, Niemczyk A, Szade J, Kapkowski M, Zenderowska P, et al Bimetallic nano-Pd/PdO/Cu system as a highly effective catalyst for the Sonogashira reaction. J Catal. 2014;313: 1–8. 10.1016/j.jcat.2014.02.008

[19] Berlman I. Handbook of fluorescence spectra of aromatic molecules 2nd ed. New York: Academic Press; 1971.

[20] Frisch MJ, Trucks GW, Schlegel HB, Scuseria GE, Robb MA, Cheeseman JR, et al Gaussian 09 Revision A.1 Gaussian Inc; 2009 Wallingford CT.

[21] Cossi M, Barone V. Solvent effect on vertical electronic transitions by the polarizable continuum model. J Chem Phys. 2000;112: 2427–2435. 10.1063/1.480808

[22] Cossi M, Barone V, Cammi R, Tomasi J. Ab initio study of solvated molecules: a new implementation of the polarizable continuum model. Chem Phys Lett. 1996;255: 327–335. 10.1016/0009-2614(96)00349-1

[23] Foresman JB, Keith TA, Wiberg KB, Snoonian J, Frisch MJ. Solvent effects. 5. Influence of cavity shape, truncation of electrostatics and electron correlation on ab initio reaction field calculations. J Phys Chem. 1996;100: 16098–16104. 10.1021/jp960488j

[24] Tomasi J, Mennucci B, Cammi R. Quantum mechanical continuum solvation models. Chem Rev. 2005;105: 2999–3093. 10.1021/cr9904009 16092826

[25] Tomasi J, Mennucci B, Cancès E. The IEF version of the PCM solvation method: an overview of a new method addressed to study molecular solutes at the QM ab initio level. J Mol Struct THEOCHEM. 1999;464: 211–226. 10.1016/S0166-1280(98)00553-3

[26] Cammi R, Corni S, Mennucci B, Tomasi J. Electronic excitation energies of molecules in solution: state specific and linear response methods for nonequilibrium continuum solvation models. J Chem Phys. 2005;122: 104513 10.1063/1.1867373 15836338

[27] Corni S, Cammi R, Mennucci B, Tomasi J. Electronic excitation energies of molecules in solution within continuum solvation models: investigating the discrepancy between state-specific and linear-response methods. J Chem Phys. 2005;123 134512 10.1063/1.2039077 16223319

[28] Dulski M, Kempa M, Kozub P, Wójcik J, Rojkiewicz M, Kuś P, et al DFT/TD-DFTstudy of solvent effect as well the substituents influence on the different features of TPP derivatives for PDT application. Spectrochim Acta A. 2013;104: 315–327. 10.1016/j.saa.2012.11.072 23274259

[29] Improta R, Barone V, Scalmani G, Frisch MJ. A state-specific polarizable continuum model time dependent density functional theory method for excited state calculations in solution. J Chem Phys. 2006;125: 054103 10.1063/1.2222364 16942199

[30] Cieslik W, Musiol R., Nycz JE, Jampilek J, Vejsova M, Wolff M, et al Contribution to investigation of antimicrobial activity of styrylquinolines. Bioorg Med Chem.2012;20: 6960–6968. 10.1016/j.bmc.2012.10.027 23159041

[31] Wu Y, Zhang Y, Zhang L. Effects of finely dispersed metallic palladium on microstructure and properties of nanocomposites produced by sol-gel technique. China Particuology. 2004;2: 19–24. 10.1016/S1672-2515(07)60015-3

[32] Yamaguchi Y, Matsubara Y, Ochi T, Wakamiya T, Yoshida ZI. How the π conjugation length affects the fluorescence emission efficiency. J Am Chem Soc. 2008;130: 13867–13869. 10.1021/ja8040493 18816053

[33] Hay PJ, Wadt WR. Ab initio effective core potentials for molecular calculations. Potentials for K to Au including the outermost core orbitals. J Chem Phys. 1985;82: 299–310. 10.1063/1.448975

[34] Wadt WR, Hay PJ. Ab initio effective core potentials for molecular calculations. Potentials for main group elements Na to Bi. J Chem Phys. 1985;82: 284–298. 10.1063/1.448800

